# Attentional blink and putative noninvasive dopamine markers: Two experiments to consolidate possible associations

**DOI:** 10.3758/s13415-019-00717-z

**Published:** 2019-08-08

**Authors:** Anne Charlotte Trutti, Zsuzsika Sjoerds, Bernhard Hommel

**Affiliations:** Cognitive Psychology Unit & Leiden Institute for Brain & Cognition, Institute of Psychology, Leiden University, Wassenaarseweg 52, 2333 AK Leiden, The Netherlands

**Keywords:** Attentional blink, Dopamine, Eye blink rate, Color vision, Mood, Metacontrol

## Abstract

Adaptive behavioral control involves a balance between top-down persistence and flexible updating of goals under changing demands. According to the metacontrol state model (MSM), this balance emerges from the interaction between the frontal and the striatal dopaminergic system. The attentional blink (AB) task has been argued to tap into the interaction between persistence and flexibility, as it reflects overpersistence—the too-exclusive allocation of attentional resources to the processing of the first of two consecutive targets. Notably, previous studies are inconclusive about the association between the AB and noninvasive proxies of dopamine including the spontaneous eye blink rate (sEBR), which allegedly assesses striatal dopamine levels. We aimed to substantiate and extend previous attempts to predict individual sizes of the AB in two separate experiments with larger sample sizes (*N* = 71 & *N* = 65) by means of noninvasive behavioral and physiological proxies of dopamine (DA), such as sEBR and mood measures, which are likely to reflect striatal dopamine levels, and color discrimination, which has been argued to tap into the frontal dopamine levels. Our findings did not confirm the prediction that AB size covaries with sEBR, mood, or color discrimination. The implications of this inconsistency with previous observations are discussed.

**Electronic supplementary material:**

The online version of this article (10.3758/s13415-019-00717-z) contains supplementary material, which is available to authorized users.

## Introduction

Human behavior is particularly flexible and adaptive, but to perform optimally in a given situation, it is necessary to find an optimal balance between environmental and endogenous goal-related contributions to persistent versus flexible behavioral control. There is increasing evidence of systematic interindividual and intraindividual differences in the degree to which people rely on endogenous top-down control and exogenous stimulus-driven contributions to control, which suggests some degree of control over the relative contributions of endogenous and exogenous sources to information processing.

The control of, and the interindividual and intraindividual differences in, persistence and flexibility has been linked to dopamine (DA; Beeler, Daw, Frazier, & Zhuang, 2010; Boulougouris, Castane, & Robbins, 2009; Colzato, Waszak, Nieuwenhuis, Posthuma, & Hommel 2010; Cools & D’Esposito 2011; Goschke & Bolte, 2014; Hommel, 2015; Klanker, Feenstra, & Denys, 2013), an essential neurotransmitter for many executive processes, such as action selection and working memory updating. It has been argued that the balance between persistence and flexibility has emerged from the interplay of two antagonistic dopaminergic systems (or of particular receptor families dominating these systems)—the mesofrontal pathway originating in the ventral tegmental area (which is assumed to promote persistence) and the nigrostriatal pathway originating in the substantia nigra (which is assumed to promote flexibility; Cools & D’Esposito, 2011; Cools, Gibbs, Miyakawa, Jagust, & D’Esposito, 2008; Cools, Ivry, & D’Esposito, 2006; Durstewitz & Seamans, 2008). This scenario fits with the important roles of the prefrontal cortex in various working memory functions, including the maintenance of action goals and other information over time (Durstewitz, Seamans, & Sejnowski, 2000), and of the striatum in regulating the updating of working memory (Cools & D’Esposito, 2011) and interrupting ongoing actions in the face of changing demands (Frank, Samanta, Moustafa & Sherman, 2007).

It has been argued that the attentional blink (AB) task (Raymond, Shapiro, & Arnell, 1992) taps into the interaction between persistence and flexibility (Colzato, Slagter, de Rover, & Hommel, 2011; Colzato, Slagter, Spapé, & Hommel, 2008; Slagter et al., 2012). The AB effect is observed when two masked (or difficult to identify) target stimuli appear in close temporal proximity: Although reporting the first target (T1) is commonly very accurate, identification of the second target (T2) is drastically impaired if it follows T1 within 100 ms to 500 ms. The original and still most widespread assumption is that the AB reflects a structural processing bottleneck, which arises from the time demands of transferring the sensory representation of a target to, and consolidating the representation in, working memory (Chun & Potter, 1995; Jolicoeur & Dell’Acqua, 1998; Vogel, Luck, & Shapiro, 1998; for a review, see Dux & Marois, 2009). ﻿DA is thought to be involved in the dynamic regulation of the contents of working memory by enabling faster working memory updating with higher striatal DA levels as compared with lower DA levels (Cools, 2011; Jongkees & Colzato, 2016; Slagter et al., 2012). In AB, the idea is that these demands are so extensive that T2 cannot be consolidated if it appears while transfer/consolidation of T1 is still ongoing. However, various observations have demonstrated that the assumed bottleneck can be partly or entirely overcome under some circumstances, such as with relaxation instructions (Olivers & Nieuwenhuis, 2005), exposure to calming aromas (Colzato, Sellaro, Rossi Paccani, & Hommel, 2014b) and flexibility-promoting meditation techniques (Colzato, Sellaro, Samara, Baas, & Hommel, 2015), by genetic predisposition (Colzato et al., 2011), or by participants having little attentional investment into T1 (Shapiro, Schmitz, Martens, Hommel, & Schnitzler, 2006). These observations are inconsistent with the assumption of a structural bottleneck, but suggest a more strategic bottleneck that relates to (presumably dopaminergic) executive control functions: Individuals who invest more endogenous attentional resources into T1 processing than necessary (i.e., those who overinvest; Olivers & Nieuwenhuis, 2005), either through disposition or because of a particular attitude or task set, are likely to miss T2, which results in a large AB, whereas individuals who invest less resources into T1 are likely to additionally process and store T2.

Matching our notion that the AB task might be a good assessment to measure a DA-related persistence–flexibility trade-off, it has been thought that the AB effect is associated with DA functioning, as assessed with positron emission tomography (PET; Slagter et al., 2012): Larger AB’s were associated with increased D2-like receptor binding in the striatum. This provides a strong incentive to use the AB task to study possible DA involvement in the flexibility–persistence trade-off. However, methods such as PET are highly invasive, expensive, and cumbersome. Noninvasive, more feasible proxies of DA would provide a solution here. Interestingly, a likely link between striatal DA and spontaneous eye blink rate (sEBR) was established by studies demonstrating differential, opposite effects of DA-agonists and DA-antagonists on sEBR (Blin, Masson, Azulay, Fondarai, & Serratrice, 1990; Strakowski & Sax, 1998; Strakowski, Sax, Setters, & Keck, 1996). Further, de novo Parkinson’s disease patients show a generally decreased sEBR (Agostino et al., 2008; Bologna, Fasano, Modugno, Fabbrini, & Berardelli, 2012; Reddy, Patel, Hodge, & Leavitt, 2013), which is reversed following dopaminergic medication (Agostino et al., 2008; Bologna et al., 2014). This suggests that sEBR could reflect a noninvasive correlate of striatal DA levels. However, other studies have refuted this interesting link between sEBR and DA (Dang et al., 2017; Kaminer, Powers, Horn, Hui, & Evinger, 2011; Sescousse et al., 2018). (For more information on the link between sEBR and DA, see the extensive literature review on sEBR and cognitive functioning by Jongkees & Colzato, 2016.)

Studies have used sEBR as a noninvasive physiological proxy of striatal DA to link the AB to striatal DA functioning (Colzato et al., 2008; Slagter & Georgopoulou, 2013). Colzato et al. (2008) found a linear relationship between sEBR and AB; however, Slagter and Georgopoulou (2013) failed to replicate this relationship between the AB and sEBR, rendering the association inconclusive. Notably, methodological differences between the Colzato et al. (2008) and Slagter and ﻿Georgopoulou (2013) studies might explain the discrepancy, such as sample size and stimulus presentation rate (Jongkees & Colzato, 2016; Shapiro, Hanslmayr, Enns, & Lleras, 2017; Slagter & ﻿Georgopoulou, 2013).

Consequently, the overall goal of the current study was to shed more light on the association between AB and sEBR by replicating Colzato et al.’s (2008) study in two, considerably larger independent data sets. Interestingly, Colzato et al. (2008) found a negative correlation between the individual size of the AB and the blink rate, showing that those with a high blink rate produced a significantly smaller AB than those with a low blink rate. Assuming that higher blinking rates reflect higher levels of striatal DA, this would suggest that more striatal DA is associated with a smaller AB.

On the one hand, the observation of a connection between flexibility-related performance and sEBR fits with findings from other tasks. For instance, Akbari Chermahini and Hommel (2010) reported that the individual sEBR predicts flexibility in divergent thinking. This finding was replicated by the same authors 2 years later (Akbari Chermahini & Hommel, 2012), who then additionally observed that the induction of positive mood increased sEBR and that the rate of this increase predicted improvement in divergent thinking. This provides convergent evidence for a role of striatal DA in flexibility-demanding performance and supports the assumption that positive-going mood is associated with increases in striatal DA.

On the other hand, however, there are remaining discrepancies in the findings. Most importantly, Colzato et al. (2008) reported a linear relationship between sEBR and the AB, whereas Akbari Chermahini and Hommel (2010, 2012) obtained nonlinear relationships between sEBR and the flexibility measures of the divergent thinking task: They followed an inverted *U* shape, so that medium blink rates were associated with best, or most flexible, performance. This latter pattern is consistent with the typical performance functions of neuromodulators and with most pharmacological interventions (especially to the degree that they target neuromodulators), but seems inconsistent with the linear function reported by Colzato et al. (2008). One reason for the inconsistency might be that Colzato et al.’s (2008) sample size was rather small (*N* = 20) compared with other studies (Akbari Chermahini & Hommel, 2010, 2012; Slagter & Georgopoulou, 2013), as was the range of blink rates (2.4–31.8; Akbari Chermahini & Hommel, 2010, 2012; Dang et al., 2017). Sample size calculations (G-Power, bivariate normal two-tailed correlation) based on their found effects size of *R*^2^ = .281 shows that for a power of (1 − β) = .95, and an alpha of α = .05, a sample size of at least *N* = 40 would be needed. Their sample size and EBR range contrasts with the larger sample size (*N* = 117) and range (~2.0 to ~48.0) of Akbari Chermahini and Hommel (2010). It is thus possible that a larger sample would reveal the possibly nonlinear nature of the relationship between sEBR and AB, and testing this possibility was indeed the first aim of the present study.

A second aim of our study relates to performance in color discrimination, which has recently been considered as a proxy for frontal DA (Colzato et al., 2014a; Jongkees, Steenbergen, & Colzato, 2017). Altered color discrimination in the blue–yellow axis (‘tritan deficit’) has been linked to DA-associated pathologies, such as Parkinson’s disease (Büttner et al., 1995; Oh et al., 2011; Pieri, Diederich, Raman, & Goetz, 2000), Huntington’s disease and Tourette syndrome (Georgiou, Bradshaw, Phillips, Bradshaw, & Chiu, 1995; Melun, Morin, Muise, & DesRosiers, 2001; Paulus et al., 1993), ADHD (Banaschewski et al., 2006; Kim et al., 2014; Spinelli et al., 2011; Tannock, Banaschewski, & Gold, 2006), and cocaine abuse (Desai, Roy, Roy, Brown, & Smelson, 1997; Hulka, Wagner, Preller, Jenni, & Quednow, 2013; Sellaro, Hommel, & Colzato, 2014). This is considered to be due to a shared dopaminergic pathway of frontal control processes and DA-driven processing in the retina (Brandies & Yehuda, 2008; Colzato et al., 2014). Indeed, moderate evidence for color discrimination as a DA proxy comes from a study on cognitive control. One study showed that performance in a response conflict task was better in participants with high color discrimination scores, and participants showing color vision impairments on the blue–yellow color axis were associated with less efficiency in handling response conflict (Colzato et al., 2014). If color discrimination truly reflects the level and/or efficiency of frontal DA, it would be interesting to see whether this measure would be related to AB performance as well. As in Colzato et al. (2014), we related general performance in color discrimination to the individual size of the AB and assessed the impact of impairments on the blue–yellow color vision axis on AB performance.

A third aim in our study relates to the finding that mood and sEBR are correlated (Akbari Chermahini & Hommel, 2012). Given the known relationship between reward and striatal DA activity (for an overview, see Haber, 2011), such a correlation is unsurprising, and the impact of positive-going mood on flexibility-heavy creativity tasks is one of the best replicated findings in the creativity literature (cf. Baas, De Dreu, & Nijstad, 2008). However, such a relationship does suggest that mood might be another interesting indicator of striatal DA, which is why we added mood measures to our study to determine whether and how they predict individual AB magnitudes.

Getting a better idea about the sensitivity of “soft measures” like sEBR, color discrimination, and mood assessments is of both theoretical and practical relevance, because other means to assess DA activity, such as PET imaging, are invasive and expensive. In the following, we present the results of our study for two independent data sets. The study was initially carried out as part of a larger project that included a battery of different tasks (Experiment 1). Hence, to substantiate results from our first experiment while controlling for effects of fatigue or ‘spillover’ of priming effects from other tasks, we collected another set of data including exclusively those instruments that were essential for this study only (Experiment 2).

## Materials and methods

We performed two independent studies, with the same testing protocol, to study the association between AB and dopamine proxies sEBR, color discrimination, and mood.

### Ethics statement

Both Experiment 1 and Experiment 2 were approved by the local ethics committee of the Institute of Psychology, Leiden University, in accordance with The Code of Ethics of the World Medical Association (Declaration of Helsinki). The participants’ written consent was obtained prior to data collection.

### Participants

#### Experiment 1

Seventy-three young, healthy adults served as participants for course credit or monetary reward. Participants were recruited from all faculties at both Leiden University and Hogeschool Leiden, The Netherlands, and were thus a sample of highly educated students. Exclusion criteria included Axis 1 psychiatric disorder (DSM-V, 2013); no clinically significant medical disease; no use of psychotropic medication; no use of glasses or contact lenses; no color blindness. Participants filled in a screening questionnaire assessing these aspects to double-check participants’ fitness for the study following data collection by means of strictly controlling for factors likely to affect neurochemistry, such as (current or recent) medication or clinical illness. For data analysis, two participants were excluded (see Table 1). One participant was excluded because the participant indicated color blindness in the screening questionnaire (which was not noticed at data collection, but was later confirmed by our own color-vision analyses), and a second participant was excluded based on the use of neurochemistry-affecting medication, thus resulting in a sample size of *N* = 71 for Experiment 1.

**Table 1:** Sample characteristic and task performance

	Experiment 1		Experiment 2	
	Mean (*SD*)	*N* (%)	Mean (*SD*)	*N* (%)
*N*		71 (100)		65 (100)
No. of excluded subjects		2		8
**Demographics**				
Age	22.21 (2.48)		19.95 (2.88) ^a^	
Gender (female)		42 (60)		56 (86.15) ^a^
**Attentional blink task**				
AB magnitude	0.1 4(0.13)		0.13 (0.16)	
Lag 1 sparing	0.23 (0.15)		0.25 (0.19)	
T1 accuracy	0.86(0.09)		0.87(0.07)	
**Eye blink rate**				
sEBR (blinks per minute)	15.63 (10.72)		14. 90(8.97)	
**Color discrimination**				
TCDS	77.70 (19.64)		77.11 (11.89)	
CCI	1.38 (0.35)		1.37 (0.21)	
Tritan deficit (Lanthony Type III)		Perfect vision: 15 (21.13) Minor errors: 22 (30.99) Major errors: 13 (18.31) Disease: 21 (29.58)		3 (4.62) 14 (21.54) 21 (32.31) 27 (41.54)
**Mood**				
Arousal at AB task ^a^	5.77 (1.51)		5.40 (1.42)	
Valence at AB task ^a^	6.64 (1.29)		6.60 (1.23)	

^a^Two data points missing

#### Experiment 2

Seventy-three young, healthy adults served as participants for course credit or monetary reward. Participants were recruited from all faculties at Leiden University, The Netherlands, and were thus a sample of highly educated students. Exclusion criteria were the same as in Experiment 1. Closer evaluation of the screening questionnaire led to the exclusion of eight participants because of various medical reasons and/or recent use of recreational drugs or medication that affect brain chemistry, resulting in a total sample size of *N* = 65 in Experiment 2 (see Table 1).

### Apparatus and stimuli

#### Procedure: Experiment 1

This experiment was carried out in the context of a larger project in which participants were tested on multiple cognitive-control tasks to study interindividual differences and consistencies across tasks. The part reported here was devoted to investigating the link between the AB and potential physiological proxies of DA, and the (thematically unrelated) findings of the other part have been published elsewhere (Mekern, Sjoerds, & Hommel, 2019). Taken altogether, participants underwent a 2-hour session in which spontaneous eye blink rate, color vision, and mood were assessed; filled in state and trait questionnaire; and performed multiple computer tasks related to cognitive control in a counterbalanced fashion.

#### Procedure: Experiment 2

In a 1-hour session, participants’ spontaneous eye blink rate, color vision, mood, and performance in the AB task was assessed.

#### Attentional blink task

The AB task was adapted from Colzato et al. (2011). Participants were asked to recognize and report two digits (T1 and T2) presented in a rapid stream of letters. A fixation cross was displayed for 2,000 ms at the start of each trial, followed by a blank interval of 250 ms. Afterwards, a rapid serial visual presentation (RSVP) stream was shown on-screen, consisting of 15 letter/digit items, each presented for 70 ms, and an interstimulus interval of 20 ms. The stream consisted of 13 distractors (letters) and two targets (digits). Letters were drawn from the alphabet, in random fashion and without replacement. Target digits were randomly drawn from the set 1–9, and distractors were letters from the Latin alphabet, excluding *I*, *O*, *S*, and *Z*, as they resemble some of the digits too closely.

Participants were instructed to report the two-digit targets they saw. The position of the first shown target, T1, in the RSVP stream was randomly varied between Positions 3, 4, and 5 in order to reduce predictability of the target onsets. The second target digit, T2, was presented directly after T1 (Lag 1), or after another two (Lag 3) or seven (Lag 8) distractors. Participants had to report both targets immediately after the RSVP by pressing the corresponding number keys; order of report was not considered. After a minimum of 18 training trials, a full experimental session lasted approximately 15 minutes and contained two testing blocks of 72 trials each (3 lags × 24 repetitions). In Experiment 1, a small subsample (*N* = 10), that was tested in the first days, performed a more elaborate version of the AB with four lags (1, 3, 5, 8). To reduce task length, and, consequently, testing time, we removed Lag 5 from the task for the remaining testing period, and hence for the rest of the sample. All participants of Experiment 2 performed the short AB task version. No differences in AB were seen between these two task versions.

All stimuli were displayed in 16-bit color on a 17-inch CRT screen with a refresh rate of 100 Hz. Participants were seated at a viewing distance of approximately 50 cm. The fixation mark (“+”) as well as all RSVP items were presented in black in the center of a gray background (RGB: 128, 128, 128). Each item was displayed in 16-point Times New Roman font.

#### Eye blink rate

Eye blinks during rest were recorded for 6 minutes to assess sEBR. Recording was done using a high-resolution Logitech C920 HD PRO webcam with a rate of around 30 FPS. Participants were placed approximately 50 cm in front of the webcam, with their heads on a chin rest, to ensure minimal movement artifacts. It was doublechecked that participants did not wear contact lenses before the start of the experiment, and were placed in a quiet room during EBR and color vision measurements. For EBR assessment, they were instructed to sit relaxed and look at a crosshair located above the webcam. They were specifically asked not to stare, but to look at the crosshair in a calm, relaxed way. They did not receive specific instructions for blinking.

Eye blinks from the recorded videos were manually counted by two independent researchers. Inconsistencies in total count (differences >5) were double-checked. Finally, sEBR was defined as the average blink rate per minute taken over 5 minutes. The first and last 30 seconds of the 6-minute recordings were excluded from EBR calculation, as this was the period the experimenter exited and reentered the room, which might have distracted participants and influenced their sEBR. Moreover, the first 30 seconds served as a short habituation period. Reliability tests suggest a time interval of 5 minutes as the standard for measuring sEBR (Doughty, 2001; Zaman & Doughty, 1997). Therefore, we calculated the average EBR over the middle 5 minutes (see Table 1 for mean sEBR in each experiment).

#### Color discrimination

Quantitative and qualitative color discrimination scores were assessed using the Lanthony Desaturated Panel D-15 test (LD-15; Lanthony, 1978). The LD-15 is an arrangement test consisting of one fixed reference cap and 15 separate moveable caps that have to be ordered sequentially based on color. The caps all contain different shades of low-saturated colors (decreased chroma 2 Munsell) with enhanced brightness (8), modified from the classic D-15 test version (Lanthony, 1978). The test was carried out without time limit, between 09:00 and 17:00, in the same order and room, and under constant lighting conditions: a daylight fluorescent lamp supplying an illumination of 1400 lux, fixed at 30 cm above the table. All other sources of illumination were turned off.

At start of the test, the caps were laid on the table in a random order, and participants were instructed to rearrange the caps by color in the order they perceived to be correct, starting from the reference cap. Participants with color perception deficiencies would have difficulties arranging the colored caps and would likely make mistakes. The correct sequence was indicated by numbers 1 through 15 written on the bottom of the caps, which the participants could not see during test performance, for scoring purposes. The participants’ arrangement of the 15 caps can be evaluated both quantitatively and qualitatively. Quantitative scoring of color discrimination was derived from a method proposed by Bowman (1982) and Geller (2001), by computing a Total Color Distance Score (TCDS), which maps the colors used in this test into a color space describing perceptual distances. The minimum score is 56.41 and is achieved when all the caps are arranged in the correct order, whereas higher scores indicate color vision deficiencies. Related to the TCDS, a Color Confusion Index (CCI) by Bowman (1982) can be calculated, which depicts a standardized score, with a minimum of 1 (perfect color discrimination). Qualitative scoring was performed following Hulka et al.’s (2013) method. The order of each participant’s caps was plotted on a template describing a hue circle based on the placement of caps in the International Commission on Illumination Color Space (Wyszecki & Stiles, 1982). Here, single cap inversions (e.g., 1-3-2-4) can be classified as minor errors or normal mistakes, whereas cap reversals spanning two or more positions are classified as major errors. Two or more major errors indicate a specific disorder. Thus, this method is useful in differentiating normal/healthy color perception from moderate to strong congenital or acquired defects in deutan (green and green weak blindness), protan (red blindness), or tritan ( blue–yellow blindness) color discrimination. Four types of acquired dyschromatopsia relying on Verriest’s classification are proposed: Type I reflects color discrimination impairment along the red–green axis; Type II reflects combined impairments of the red–green and blue–yellow axis; Type III indicates blue–yellow axis impairments; and Type IV is diagnosed when no clear pattern can be determined (see Table 1 for classification of participants in Experiment 1 and Experiment 2). Considering the putative modulation of dopaminergic receptors in the blue–yellow axis (Brandies & Yehuda, 2008; Colzato et al. 2014; Jongkees et al., 2017), we focused our qualitative analyses on intact as well as impaired color discrimination performance in this specific axis.

#### Mood assessment

Mood data were assessed using the affect grid (Russell, Weiss, & Mendelsohn, 1989) after sEBR recording and before the AB task. The grid employs a 9 × 9 matrix, resembling a two-dimensional 9-point Likert scale. Participants are instructed to place an *X* in one of the 81 cells of the grid. The location of the *X* indicated the participant’s affective state within the two-dimensional space defined by hedonic tone and activation. Thus, the scale provides two scores: one for valence and one for arousal. Along the horizontal axis, valence is scored, from left to right, from *very unpleasant* (1) to *very pleasant* (9). Along the vertical axis, arousal is scored, from bottom to top, from *very sleepy* (1) to *highly aroused* (9).

### Statistical analyses

All analyses were carried out in the analysis software R (R Core Team, 2014; Version 1.0.153) using a critical alpha level of *p* = .05 if not stated otherwise. We first tested our assumptions of normality by carrying out the Shapiro–Wilk test from the R core package *stats*.

To investigate the presence of an attentional blink, separate repeated-measures ANOVAs for T1 accuracy and T2|T1 accuracy were conducted, with lag as a within-subjects factor with three levels (Lag 1, Lag 3, and Lag 8). The ANOVAs (described below) were carried out using ezANOVA from the R package *ez* (Version 4.4-0). AB magnitude computation was based on Colzato et al. (2008) and Slagter and Georgopoulou (2013): ﻿T2|T1 at Lag 8 ﻿minus the minimum of T2|T1 at Lag 3 and at Lag 5. Since our task version was shorter and did not have a Lag 5 for the majority of participants, we adjusted the computation to T2|T1 at Lag 8 ﻿minus T2|T1 at Lag 3.

To investigate the relationship between AB magnitude and markers of DA, sEBR, and quantitative color discrimination (CCI) scores, two analysis steps were carried out. In a first step, sEBR analyses from Colzato et al. (2008) were directly replicated. Given the incoherent findings on the relationship between sEBR and AB size found in previous studies, we additionally performed Bayesian correlation tests to assess the strength of evidence of our results on the relationship between AB and sEBR. Bayesian analyses were performed in JASP (2018; Version 0.9). In a second step, polynomial regressions on sEBR and CCI were carried out.

Following Colzato et al. (2008), we performed separate repeated-measures ANOVAs for T1 and T2|T1 accuracy, with an EBR group factor based on a median split. Experiment 1: low EBR group (36 participants, 1.8–15.2 score), high EBR group (35 participants, 15.4–63.4 score); Experiment 2: low EBR group (32 participants ﻿3.4–12.4 score), high EBR group (33 participants ﻿12.5–53.6 score). Spearman correlation tests were performed to investigate replicability of the link between AB and sEBR, as was found in Colzato et al. (2008).

In the polynomial regressions assessing the association between AB and dopamine proxies, for both independent variables sEBR and CCI, regression models including linear as well as quadratic terms were calculated, and models were selected based on model comparison using the *anova* function from the R package *car* and, if one model did not outperform the other, models were selected based on the smallest BIC score.

To investigate the association between the specific blue–yellow axis characteristics and AB size, a nonparametric analysis of variance (Kruskal–Wallis rank sum test) was performed, given the relatively small group sizes and nonparametric response variable. The sample was divided into four ordinal groups according to the qualitative scoring on the blue–yellow axis: perfect vision, minor errors, major errors, disorder. As with the polynomial regression, AB was entered as the dependent variable and color discrimination group as independent the variable. The test was carried out using R software package *stats* from the Comprehensive R Archive Network (http://cran.r-project.org/web/packages).

To explore the relationship between AB magnitude and mood, the same polynomial regressions that were used to investigate relationship between AB and the two DA markers were carried out for both mood axes, arousal and valence, independently. Again, model selection was based on model performance and/or smallest BIC.

## Results

### Attentional blink task performance

﻿Separate ANOVAs for T1 and T2|T1 accuracy data were carried out, with lag (1, 3, 8) as a within-subjects factor. T2|T1 accuracy was computed based only on trials in which the T1 was reported correctly.

#### Experiment 1

**Fig. 1:**
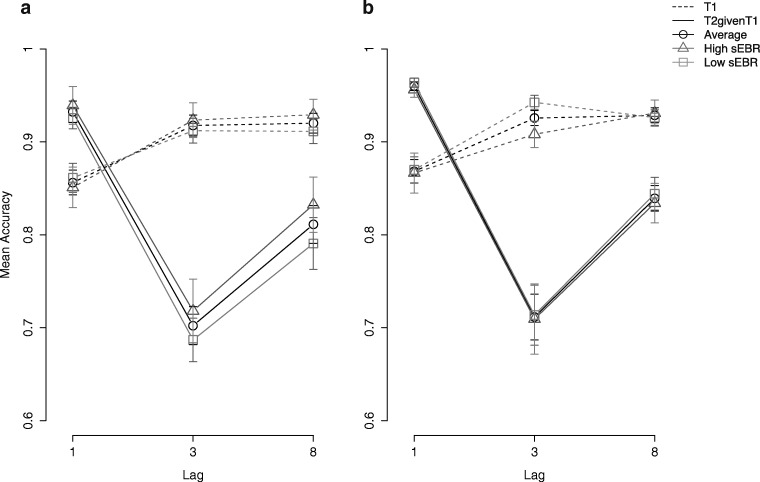
Replication of the AB effect in a short version of the AB task with three lags for the two data sets and for median split EBR groups, respectively, for Experiment 1 (**a**) and Experiment 2 (**b**). T1 performance (dashed line) and T2 performance given T1 correct (T2|T1) (solid line) shown separately for each lag and high versus low eye-blinkers and the average across all participants

Mauchly’s test of sphericity indicated that the assumption of sphericity had been violated for the repeated-measures ANOVA model on T1 accuracy data (*p* = .001). Accordingly, the corrected *p* values and *df* values (Greenhouse–Geisser epsilon correction) are reported. The repeated-measures ANOVA on T1 accuracy showed a statistically significant main effect of lag on accuracy, *F*(2, 140) = 42.36, *p*_*gg*_< .001. This lag effect was replicated in the repeated-measures ANOVA for T2|T1 accuracy data, *F*(2, 140) = 99.44 , *p* < .001. Taken together, our data show the classical AB effect (See Fig. 1a).

#### Experiment 2

Mauchly’s test of sphericity indicated that the assumption of sphericity had been violated for the repeated-measures ANOVA model on both T1 accuracy (*p* = .014) and T2|T1 accuracy data (*p* < .001). Accordingly, the corrected *p* values and *df* values (Greenhouse–Geisser epsilon correction) are reported. For T1 accuracy, the repeated-measures ANOVA again revealed a statistically significant main effect of lag, *F*(2, 128) = 30.7, *p*_*gg*_ < .001 , as did it for T2|T1 accuracy data, *F*(2, 128) = 82.82, *p*_*gg*_ < .001. Hence, also our data from Experiment 2 shows the classical AB effect (see Fig. 1b).

### Attentional blink and dopamine markers

#### AB and sEBR association

##### Experiment 1

The sEBR ranged from 3.4 to 53.6, with a mean of 14.09 (*SD* = 8.97). Mauchly’s test of sphericity indicated that the assumption of sphericity had been violated for the AB–sEBR associating repeated-measures ANOVA model on T1 accuracy, for both the lag (*p* = .002) and the interaction between sEBR group and lag (*p* = .002). Accordingly, the corrected *p* values and *df* values (Greenhouse–Geisser epsilon correction) are reported.

The repeated-measures ANOVA on T1 accuracy showed no statistically significant main effect of sEBR group on T1 accuracy, *F*(1, 69) = 0.08, *p* = .78, and no interaction between sEBR group and lag, *F*(2, 138) = 1.79, *p*_*gg*_ = .17. There was a significant main effect of lag, *F*(2, 138) = 42.87, *p*_*gg*_ < .001; see Fig. 1a). The repeated-measures ANOVA on T2|T1 accuracy also did not reveal a statistically significant main effect of sEBR group, *F*(2, 69) = 0.80, *p* = .350, and no interaction between sEBR group and lag, *F*(2, 138) = 0.26, *p* = .685, but a significant main effect of lag, *F*(2, 138) = 98.30, *p* < .001 (see Fig. 1a).

To test replicability of the main finding from Colzato et al. (2008) on the association between sEBR and the size of the AB, we carried out correlation tests with an adjusted p-value of *p* = .0125, in order to control for multiple comparisons. Given the nonparametric nature of the data, a Spearman correlation test was applied in both data sets. ﻿Against the prediction, sEBR did not correlate with AB size (*r*_s_ = .048, *p* = .685). Further, it did not correlate with Lag 1 sparing (*r*_*s*_ = -.081, *p* = .500), or mean T1 (*r*_*s*_ = -.151, *p *= .209), and T2|T1 accuracy) (*r*_*s*_ = -.24,* p* = .044). In addition, the data were examined by estimating a Bayes factor using BIC (Wagenmakers, 2007). This compares the fit of the data under the null hypothesis, compared with the alternative hypothesis. The Bayesian, undirected correlation test (rho) with an uninformative prior (beta = 1) estimated Bayes factor (﻿BF_01_ = 6.07) suggested moderate evidence in favor of the null hypothesis. More precisely, the estimated Bayes factor suggested the data are 6.07 times more likely under the null hypotheses—namely, that AB and sEBR are not correlated.

##### Experiment 2

Mauchly’s test of sphericity indicated that the assumption of sphericity had been violated for the repeated measures ANOVA model on T1 accuracy, for both the lag (*p* < .001) and the interaction between sEBR group and lag (*p* < .001). Accordingly, the corrected *p* values and *df* values (Greenhouse–Geisser epsilon correction) are reported. sEBR ranged from 1.8 to 63.4 with a mean of 15.03 (*SD* = 9.15).

There was no main effect of sEBR group on T1 accuracy, *F*(1, 63) = 0.41, *p* = .523, and no interaction between sEBR group and lag, *F*(2, 126) = 2.91, *p*_*gg*_ = .070. Again, there was a main effect of lag, *F*(2, 126) = 31.62 , *p*_*gg*_ < .001 (see Fig. 1b). The separate ANOVA on T2|T1 accuracy also did not reveal a main effect of sEBR group, *F*(2, 63) = 0.09, *p* = .766, no interaction between sEBR group and lag, *F*(2, 126) = 0.01, *p* = .990, only a main effect of lag, *F*(2, 126) = 81.54, *p* < .001 (Fig. 1b).

As for Experiment 1, nonparametric correlation tests, with a corrected p-value for multiple comparisons, again did not reveal evidence for a relationship between sEBR and AB size (*r*_s_ = –.025, *p* = .843), sEBR and Lag 1 sparing (*r*_s_ = −.021, *p* = .867), sEBR and mean T1 (*r*_s_ = .078, *p* = .536), and sEBR and mean T2|T1 accuracy (*r*_s_ = .038, *p =* .762) in Experiment 2. The Bayesian, undirected correlation test (rho) with an uninformative prior (beta = 1) estimated Bayes factor (﻿BF_01_ = 6.46) suggested moderate evidence in favor of the null hypothesis. In other words, the estimated Bayes factor suggested the data are 6.46 times more likely under the null hypotheses, indicating again that sEBR and AB are not correlated.

#### Polynomial regressions on DA markers

Polynomial regression analysis was carried out to test if the DA markers, sEBR and quantitative CD (CCI), respectively, significantly predicted participants’ size of the AB. First, a linear regression model, including a single linear term for the predictors sEBR and CCI, respectively, was used. In a second step, the linear model was compared with a second, quadratic model, which included an additional quadratic term for the predictor. Predictors in the regression model were mean centered for analysis. All predictor variables were mean centered before being added to the regression model.

#### AB and sEBR

##### Experiment 1

**Fig. 2:**
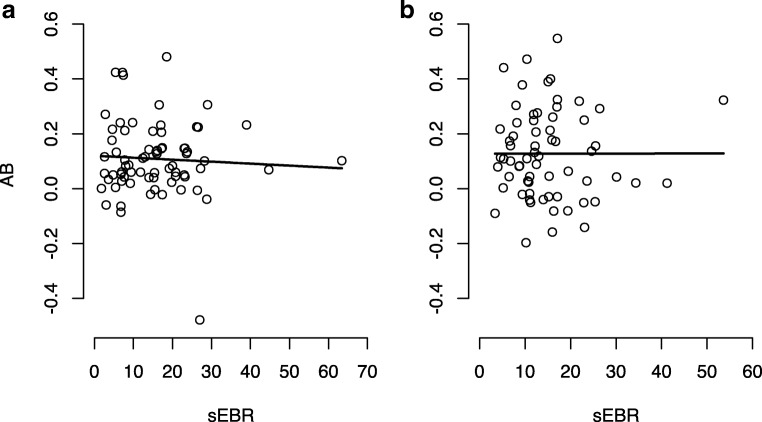
No indication of a relationship between sEBR and size of the AB. **a** Experiment 1, *F*(1, 69) = .22*, p* = .641, *R*^2^ = .003. **b** Experiment 2, *F*(1, 63) = .00, *p* = .993, *R*^2^ = −.016. Please note the removal of outliers did not change the outcome of the analysis nor did it improve model fit

As expected from the nonsignificant correlation we report above, polynomial regression did not reveal a significant linear relationship between AB and sEBR, *F*(1, 69) = 0.22, *p* = .641, *R*^2^ = .003 (Fig. 2a). The quadratic model did not outperform the linear model, indicating no (curvi) linear relationship between the variables. Hence, sEBR did not significantly predict the size of the AB (β = −.0007, *p* = .641), in either a linear or in a quadratic fashion. It should be noted that there were no influential observations in the regression model, and removal of outliers did not result in an improvement of the model.

##### Experiment 2

Again, polynomial regression did not indicate any (linear or quadratic) relationship between AB and sEBR, *F*(1, 63) < 0.001, *p* = .993, *R*^2^ = −.016 (Fig. 2b). Hence, sEBR did not significantly predict the size of the AB (β < 0.001, *p* = .993). It should be noted that removal of influential observations in the regression model resulted in improvement of the model. Yet the model did not become significant, and *R*^2^ remained around zero; therefore, we report the regression results before removal of observations (see the Supplementary Materials for more information on regression model selection).

#### AB and color discrimination

##### Experiment 1

**Fig. 3:**
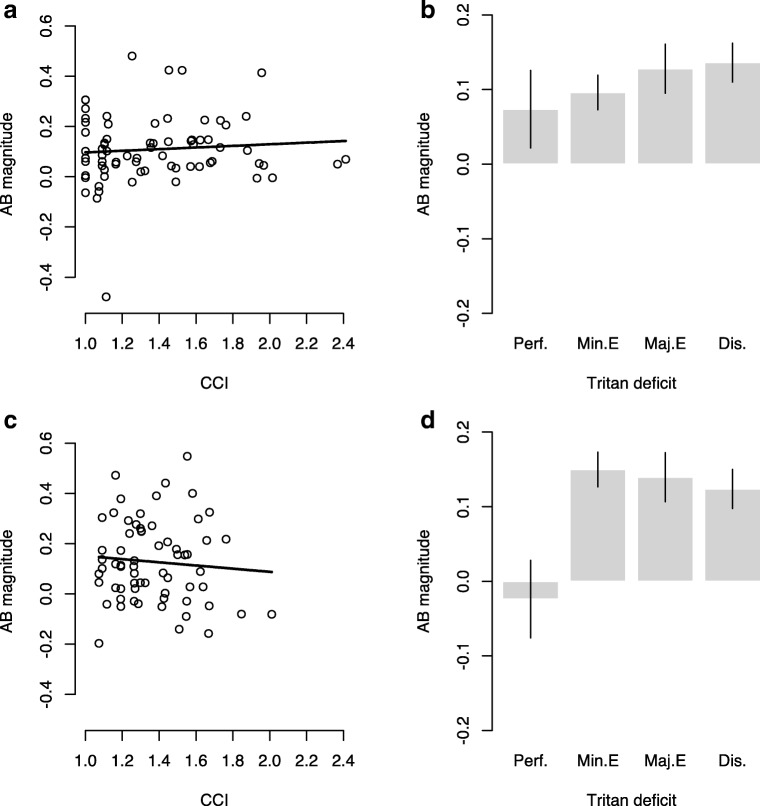
AB magnitude as a function of color discrimination performance. **a, c** Data does not indicate a relationship between the quantitative color discrimination variable (CCI) and AB size, Experiment 1: *F*(1, 69) = 0.46, *p* = .499, *R*^2^ = .01; Experiment 2: *F*(1, 62) = 1.11, *p* = .296, *R*^2^ = .002. **b, d** Accuracy in color discrimination on the blue–yellow axis (‘tritan deficit’) does not explain AB magnitude differences, Experiment 1: χ^2^(3) = 0.744, *p* = .53; Experiment 2: *χ*^2^(3) = 2.582, *p* = .461. Color discrimination on the blue–yellow axis was qualitatively assessed and participants were split up in groups accordingly. Perf. indicates perfect color discrimination; Min. E and Maj. E indicate minor and major errors in color discrimination, respectively, and Dis. reflects color discrimination on the blue–yellow axis that is classified as a disorder. Please note the removal of outliers did not change the outcome of the analysis nor did it improve model fit

Polynomial regressions on CCI and AB did not reveal any relationship between the variables, *F*(1, 69) = 0.46, *p* = .499, *R*^2^ = .01 (see Fig. 3a). This indicates that color discrimination does not significantly predict the size of the AB (β = 0.032, *p* = .499).

Nonparametric factor analysis using the Kruskal–Wallis test, with blue–yellow color discrimination quality as a between-subjects variable, failed to reveal an association between AB and blue–yellow color discrimination quality, χ^2^(3) = 0.744, *p* = .53 (see Fig. 3b).

##### Experiment 2

Polynomial regression analysis on the relationship between CCI and AB did not reveal any relationship between the variables, *F*(1, 62) = 0.03, *p*= ..870, *R*^2^ = .02 (see Fig. 3c); therefore, as in Experiment 1, color discrimination does not predict the size of the AB (β = −0.02, *p* = .870). Note that one observation with high leverage (Cook’s distance = 0.11 > 4 × mean Cook’s distance) has been excluded from the analysis because the model indicated a curvilinear relationship due to an almost significant quadratic CCI term (β = −.634, *p* = .089). However, the quadratic model was not significant, *F*(2, 62) = 1.72, *p* = .188, *R*^2^ = .02, and removal of the observations removed the (nonsignificant) tendency toward a quadratic effect.

Nonparametric factor analysis, with blue–yellow color discrimination quality as a between-subjects variable, again failed to show an association between AB and blue–yellow color discrimination quality, as in Experiment 1, *χ*^2^(3) = 1.0, *p* = .398 (see Fig. 3d).

### AB and mood

Polynomial regression analyses were carried out to test whether the AB magnitude is predicted by mood-related arousal and valence.

#### Experiment 1

Analyses show that arousal before AB assessment does not predict the AB, *F*(1, 67) = 0.09, *p* = .761,* R*^*2*^ = .001. Regression analysis for valence revealed that, in accordance with arousal, the size of the AB is not significantly predicted by valence, *F*(1, 67) = 3.0, *p* = .088, *R*^*2*^ = .04.

#### Experiment 2

As in Experiment 1, both arousal and valence in Experiment 2 failed to significantly predict the AB—arousal: *F*(1, 63) = 0.74, *p* = .391; valence: *F*(1, 63) = 2.87, *p* = .095. Yet removal of one influential observation (Cook’s distance = 0.16 > 4 × mean Cook’s distance) in the regression model revealed a significant negative linear relationship between valence and the size of the AB, *F*(1, 62) = 4.75, *p* = .033.

## Discussion

This study focused on the impact of striatal dopamine (DA), as putatively assessed by noninvasive physiological and behavioral markers, on the allocation of attentional resources in the attentional blink (AB) task. We set out to replicate, in two separate high-powered experiments, previous studies (Colzato et al., 2008; Slagter & Georgopoulou, 2013) that showed a disagreement on the association between a proxy of striatal DA levels (spontaneous eyeblink rate; sEBR) and the AB effect. Within this overall goal, we had three specific aims. First, we were interested to see whether an apparent inconsistency between the linear function relating sEBR and AB reported by Colzato et al. (2008) and the nonlinear functions relating sEBR to flexibility-heavy creativity tasks reported by Akbari Chermahini and Hommel (2010, 2012) could be resolved. We considered the possibility that a larger sample than in the original study by Colzato and colleagues might reveal a more nonlinear function. We expected that best performance (i.e., the smallest AB) goes along with medium blink rates, which fits with the inverted *U* shape reported by Akbari Chermahini and Hommel (2010, 2012)—note that in these studies good performance (or high flexibility) was indicated by high values on the *y-*axis whereas the opposite was the case in the present study. Yet we were not able to find a linear, or the hypothesized *U*-shaped, relationship between AB and sEBR. Therefore, our findings are not in line with multiple accounts that report either linear (Colzato et al., 2008) or *U*-shaped relationships between dopamine, as measured with multiple methods, and cognitive performance (Arnsten, 1997; Cools & D’Esposito, 2011; Gjedde, Kumakura, Cumming, Linnet, & Moller, 2010; Williams & Castner, 2006). Nonlinear functions of the inverted *U* shape kind are more typical for the impact of neuromodulators on human behavior and fit better with the available evidence of interventions targeting or affecting DA-driven processes than the first-reported linear function.

Our second question was whether color discrimination, a possible marker of frontal DA (Colzato et al., 2014; Jongkees et al., 2017), might also be related to the individual size of the AB. If so, this would imply that the dopaminergic impact on AB is not restricted to striatal DA but might also comprise other dopaminergic sources. However, both the quantitative and qualitative analyses revealed no systematic connection between color discrimination and AB. Note that not much is known about the relationship between color discrimination and frontal DA, and it is certainly possible that other, more sensitive measures of frontal DA do indicate a systematic connection. For the time being, however, we conclude that AB does not seem to be sensitive to the factors responsible for color-discrimination performance.

Our third aim was whether mood, which often has been related to striatal DA, could be shown to affect AB as well. We distinguished between the arousal and valence aspect of mood. Experiment 1 did not reveal a significant relationship between mood and AB size, and Experiment 2 yielded significant relationships for both valence and arousal after the exclusion of one influential outlier. It might be tempting to relate the latter observation to the fact that in Experiment 1 the valence–AB correlation was close to significant (*p* = .063), but closer inspection reveals that this correlation was positive while the significant correlations in Experiment 2 were negative. Hence, even the few significant effects in our study turned out to be nonreplicable.

The same holds true for the relationship between mood and sEBR: The negative correlations for both valence and arousal observed in Experiment 1 could not be replicated in Experiment 2, where the correlations were far from significant. Yet, when pooling the data, the results of mood and sEBR association further attest the findings of Experiment 1, with arousal being negatively associated with sEBR. Taken altogether, the mood-related findings provide weak and unsystematic support for the assumption of a connection between mood and striatal DA levels. The fact that none of these findings was really replicable suggests that important moderators underlying this connection are not yet understood.

Taken altogether, our findings do not provide support for any systematic relationship between the size of the AB and sEBR, color vision, or mood. Note that the prediction of such relationship hinges on a number of assumptions, not all of which are necessarily challenged by our findings. One possibility is that AB does relate to striatal DA (Slagter et al., 2012), but none of the three measures is sufficiently sensitive to striatal DA levels. For instance, it might be that sEBR is a reliable predictor of the *phasic changes* in striatal DA levels—as, for instance, indicated by the study of Akbari Chermahini and Hommel (2012)—but not of tonic levels, as assumed by Colzato et al. (2008). Indeed, Akbari Chermahini and Hommel found systematic and significant correlations between changes in sEBR and changes in mood, and a high predictability of the changes in creative performance by these changes, whereas the base levels of sEBR and mood failed to explain aspects of creative performance. Tonic striatal DA levels and phasic changes therein are known to be related, but the kind of relationship is not yet fully understood (Guthrie, Myers, & Gluck, 2009; Zhang, Doyon, Clark, Phillips, & Dani, 2009). It might thus be that sEBR only indicates phasic changes in a reliable fashion, but, depending on not yet fully understood aspects of the task, the task environment, and/or the sample, they may also pick up important, performance-predictive aspects of the tonic DA level. Alternatively, it is possible that it is actually tonic DA levels that actually matter for task performance. The sEBR may only be sensitive to phasic DA changes, but, depending on the tonic level and/or other not yet fully understood sample characteristics, phasic changes and tonic levels may vary in the degree to which they are correlated. Accordingly, under some circumstances (e.g., within some range of the tonic level), sEBR may reflect (i.e., correlate with) the tonic level more than under other circumstances, and similar considerations may hold for mood and color vision.

Another possibility, is that sEBR does not relate to AB in a way that more direct striatal DA measures relate to AB, and is therefore not correlated. When AB size relates to D2-like receptor availability as measured in Slagter et al. (2012), sEBR might reflect some alternative striatal DA attribute, for example DA ﻿transporter density (Sescousse et al., 2018). In fact, findings are incoherent when it comes to the associations between sEBR and DA ﻿receptor availability (Dang et al., 2017; Groman et al., 2014; Sescousse et al., 2018).

An alternative explanation for the lack of support for any systematic relationship between the size of the AB and the observed variables, is that ﻿the limiting factors of working memory consolidation appear influenced by specific brain oscillations (Shapiro et al., 2017). In spite of the theoretical accounts of the AB that link it to DA, a recent study has shown that AB is frequency specific: The frequency of stimulus presentation in a rapid stream of stimuli affects the AB magnitude by means of triggering oscillatory frequencies in the brain that are more or less involved with the processing of visual attention and conscious awareness (Shapiro et al., 2017). The significance of oscillations in the AB was further substantiated by another recent study that revealed the importance of the coordination of saccadic eye movements with oscillatory alpha frequencies for successful memory encoding (Staudigl, Hartl, Noachtar, Doeller, & Jensen, 2017). Instead of time between target stimuli (the lag) being the only crucial factor for memory transfer and consolidation into working memory, the frequency of stimulus presentation influences working memory capacities in the AB task. In nonhuman primates, endogenous midfrequency oscillations between 19 and 38 Hz in the ﻿primary visual cortex are linked to dopaminergic neuromodulation (Zaldivar, Goense, Lowe, Logothetis, & Panzeri, 2018). How (striatal) DA is involved in modulating oscillatory frequency bands in humans is still unclear, as is the complicated relationship between dopaminergic modulation and working memory (Motley, 2018). This altogether renders claims on the relationship between AB and DA, and DA and brain oscillations, fairly speculative. Nevertheless, shedding light on the relationship between AB, DA, and brain oscillations in future research would provide the literature with valuable information about the underlying mechanisms of AB.

Another possible explanation is that irrespective of whether sEBR is or is not a direct reflection of striatal DA activity, AB does not rely on striatal DA. For instance, the available evidence suggesting that the size of AB reflects a cognitive strategy rather than a structural bottleneck might be taken to point to the frontal dopaminergic pathway and prefrontal cortex as the responsible agent. If so, it may well be that sEBR is a reliable indicator of striatal DA activity. Given the widespread assumption that frontal and striatal control networks interact (Cools, 2011), it may even be that frontal and striatal DA levels are correlated under some, not yet fully understood circumstances, so that sometimes sEBR does correlate with task performance, even though it is not striatal but frontal DA that is actually involved in the relevant control operations.

Finally, we note that sEBRs were measured differently than in the study of Colzato et al. (2008)—namely, by means of a camera rather than EOG. It is possible that applying these methods leads to systematically different outcomes and/or two different degrees of noise in the data. We are currently in the process of systematically assessing the reliability of these measurement instruments in our lab.

These speculations suggest that we need more, and more systematic, insights into the relationship between tonic and phasic DA activity, and the relationship between this activity and sEBR, mood, and color vision. At this point, however, there are reasons to believe that sEBR, mood, and color vision cannot be considered to reflect striatal DA activity in any one-to-one fashion.

## Electronic supplementary material

ESM 1 (PDF 293 kb)
