# Supplementary material for: Attentional blink and putative noninvasive dopamine markers: Two experiments to consolidate possible associations
Source: Cogn Affect Behav Neurosci. 2019 Aug 8;19(6):1444–57. doi: 10.3758/s13415-019-00717-z (PMC6861702; doi:10.3758/s13415-019-00717-z)
Supplement: Supplementary file 1 — (PDF 293 kb) [file 13415_2019_717_MOESM1_ESM.pdf]

Appendix: Supplementary Material

Table of Contents

1. Full regression model 1
2. Analysis on pooled data (N=136) 2
3. AB – sEBR: experiment 2 – removal of influential observations 7
4. AB – sEBR: analysis with adjusted sEBR range 8
5. AB – sEBR: other AB measures (as in Willems et al., 2015) 9
6. AB: reversal ratio influences, an additional explorative analysis 9
7. sEBR and mood 12

1. Full regression model

Multiple regression analysis was carried out to test whether the AB magnitude is predicted by all (continuous) explanatory variables tested, namely sEBR, color discrimination (CCI), and mood variables arousal and valence. Since, in the individual regression models did not reveal curvilinear relationships between the variables, the predictors are added as single, linear predictors.

Experiment 1

Analyses show that the full model, including all continuous predictors, does not predict AB size ($F(15,53)$ =1.212, p= .284, R^2 = .04). See table S1 for the beta estimates of the individual predictor terms.

Experiment 2

Analyses show that the full model, including all continuous predictors, does not predict AB size ($F(15,49)$ =1.302, p= .238, R^2 = .07). See table S2 for the beta estimates of the individual predictor terms.

Full regression model analyses on both data sets combined can be found in the next paragraph (2.2.3. Multiple regression model on DA markers)

Table S1. *Regression results using AB size as the criterion on data of experiment 1.*

Predictor	b	p-value
(Intercept)	-0.84	.862
sEBR	-0.01	.954
CCI	0.24	.948
arousal_ab	0.07	.936
valence_ab	0.39	.591
sEBR:CCI	0.04	.819
sEBR:arousal_ab	-0.00	.951
CCI:arousal_ab	0.03	.964
sEBR:valence_ab	-0.01	.822
CCI:valence_ab	-0.21	.708
arousal_ab:valence_ab	-0.05	.645
sEBR:CCI:arousal_ab	-0.01	.872
sEBR:CCI:valence_ab	0.00	.964
sEBR:arousal_ab:valence_ab	0.00	.714
CCI:arousal_ab:valence_ab	0.03	.758
sEBR:CCI:arousal_ab:valence_ab	-0.00	.884

Table S2. *Regression results using AB size as the criterion on data of experiment 2.*

Predictor	b	p-value
(Intercept)	-8.96	.402
sEBR	0.29	.721
CCI	4.03	.580
arousal_ab	1.54	.388
valence_ab	1.12	.474
sEBR:CCI	-0.02	.975
sEBR:arousal_ab	-0.03	.802
CCI:arousal_ab	-0.66	.590
sEBR:valence_ab	-0.03	.799
CCI:valence_ab	-0.45	.683
arousal_ab:valence_ab	-0.19	.465
sEBR:CCI:arousal_ab	-0.01	.943
sEBR:CCI:valence_ab	-0.01	.949
sEBR:arousal_ab:valence_ab	0.00	.863
CCI:arousal_ab:valence_ab	0.07	.688
sEBR:CCI:arousal_ab:valence_ab	0.00	.898

2. Analysis on pooled data (N=136)

To explore if a larger sample size, containing higher power, would confirm our findings in the two separate experiments, we additionally pooled the data of the two experiments and repeated the analyses on this larger dataset of 136 participants. It should be noted, however, that pooling the

datasets into one larger dataset has, apart from the profit of increasing power, some significant caveats, and was therefore not our main focus. The two experiments were performed with minimally a year apart, by different experimenters. Moreover, experiment 1 was part of a larger study, assessing additional tasks (published elsewhere: Mekern, Sjoerds & Hommel, 2019), whereas experiment 2 was specifically performed to replicate the AB-EBR effects in experiment 1, and was therefore entirely designed around this goal. Also, some differences in environment (labs) and hardware (computers / screens) are present between the experiments. Results of pooled datasets that are so different in many aspects, therefore should be approached with caution.

2.1. Attentional Blink task performance

Separate ANOVAs for T1 and T2|T1 accuracy data respectively, were carried out with lag (1, 3, 8) as within-subjects factor. T2|T1 accuracy was computed based on only those trials in which the T1 was reported correctly.

Mauchly's Test of Sphericity indicated that the assumption of sphericity had been violated for the repeated measures ANOVA model on T1 accuracy data ($p < .001$). Accordingly, the corrected P-values and DF-values (Greenhouse-Geisser epsilon correction) are reported. The repeated-measures ANOVA on T1 accuracy showed a statistically significant main effect of lag on accuracy ($F(2, 270) = 73.03$, $p_{gg}<.001$). This lag effect was replicated in the repeated-measures ANOVA for T2|T1 accuracy data ($F(2, 270) = 181.7$, $p<.001$).

2.2. Attentional Blink and dopamine markers

2.2.1. Analysis replication of Colzato et al. 2008

sEBR ranged from 1.8 to 63.4 with a mean of 15.28 (SD = 9.89). Mauchly's Test of Sphericity indicated that the assumption of sphericity had been violated for the AB-sEBR associating repeated measures ANOVA model on T1 accuracy, for both the lag ($p < .001$) and the interaction between sEBR group and lag ($p < .001$). Accordingly, the corrected P-values and DF-values (Greenhouse-Geisser epsilon correction) are reported.

The repeated-measures ANOVA on T1 accuracy showed no statistically significant main effect of sEBR group on T1 accuracy ($F(1, 134) = 0.073$, $p=.787$), nor an interaction between sEBR group and lag ($F(2, 268) = 3.01$, $p_{gg}=.069$). There was a significant main effect of lag ($F(2, 268) =$

74.12, p_{gg}<.001). The repeated-measures ANOVA on T2|T1 accuracy also did not reveal a statistically significant main effect of sEBR group (F(2, 134)= 0.21, p=.645), nor an interaction between sEBR group and lag (F(2, 268) = 0.09, p_{gg}=.91), but a significant main effect of lag (F(2, 268) = 180.48, p_{gg}<.001; Figure S1).

In order to test replicability of the main finding from Colzato et al. (2008) on the association between sEBR and the size of the AB, we carried out correlation tests. Given the non-parametric nature of the data spearman correlation test was applied in both data sets. Against the prediction, EBR did not (negatively) correlate with AB size (r_s = −.005, p= .946). Further, it did not correlate with Lag-1 sparing (r_s = .02, p= .789), or mean T1 (r_s = −.03, p= .693) and T2|T1 accuracy (r_s = −.11, p= .204). In addition, the data were examined by estimating a Bayes factor using Bayesian Information Criteria (Wagenmakers, 2007). This compares the fit of the data under the null hypothesis, compared to the alternative hypothesis. The Bayesian, undirected correlation test (rho) with an uninformative prior (beta = 1) estimated Bayes factor (BF_{01} = 8.76) suggested moderate evidence in favor of the null hypothesis. More precisely, the estimated Bayes factor suggested the data are 8.76 times more likely under the null hypotheses, namely that AB and sEBR are not correlated.

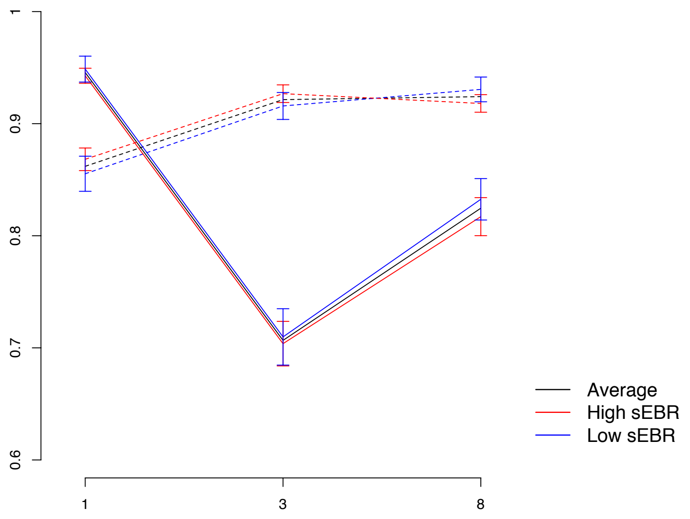

Figure S1. Replication of the AB effect in a short version of the AB task with three lags for the two data sets combined and for median split EBR groups respectively. T1 performance (dashed line) and T2 performance given T1 correct (T2|T1) (solid line) shown separately for each lag and high versus low eye-blinkers and the average across all participants.

2.2.2. Polynomial regressions on DA markers

Polynomial regression analysis was carried out to test if the DA markers, sEBR and quantitative CD (CCI) respectively, significantly predicted participants' size of the AB. First, a linear regression model, including a single linear term for the predictors sEBR and CCI respectively, was used. In a second step, the linear model was compared to a second, quadratic model which included an additional quadratic term for the predictor. All predictor variables were mean centered before added to the regression model.

2.2.2.1. AB and sEBR

As expected from the non-significant correlation we report above, polynomial regression did not reveal a significant linear relationship between AB and sEBR ($F(1,134)=0.13$, $p=.722$, $R^2=-.007$; Figure S2). The quadratic model did not outperform the linear model, indicating no (curvi)linear relationship between the variables. Hence, sEBR did not significantly predict the size of the AB ($\beta = -.0004$, $p=.722$), neither in a linear, nor in a quadratic fashion.

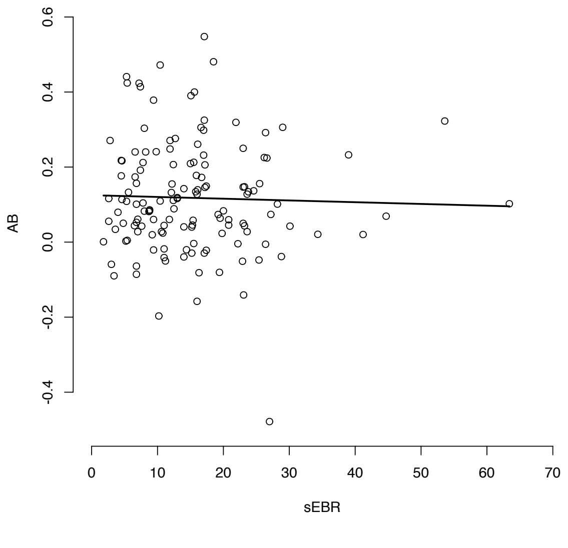

Figure S2. No indication of a relationship between sEBR and size of the AB. A) Experiment 1 ($F(1,69)=.22$, $p=.641$, $R^2=0.003$). B) Experiment 2 ($F(1,63)=.00$, $p=.993$, $R^2=-0.016$). Please note the removal of outliers did not change the outcome of the analysis nor did it improve model fit.

2.2.2.2. AB and color discrimination

Polynomial regressions on CCI and AB did not reveal any relationship between the variables ($F(1,134)$ =0.03, p= .864, R^2 = -.007; Figure S3A) This indicates that color discrimination does not significantly predict the size of the AB (β = 0.008, p=.864).

Non-parametric factor analysis using the Kruskal-Wallis test with blue-yellow color discrimination quality as between-subject variable failed to reveal an association between AB and blue-yellow color discrimination quality ($\chi^2(3)$ = 1.23, p = .301; Figure S3B).

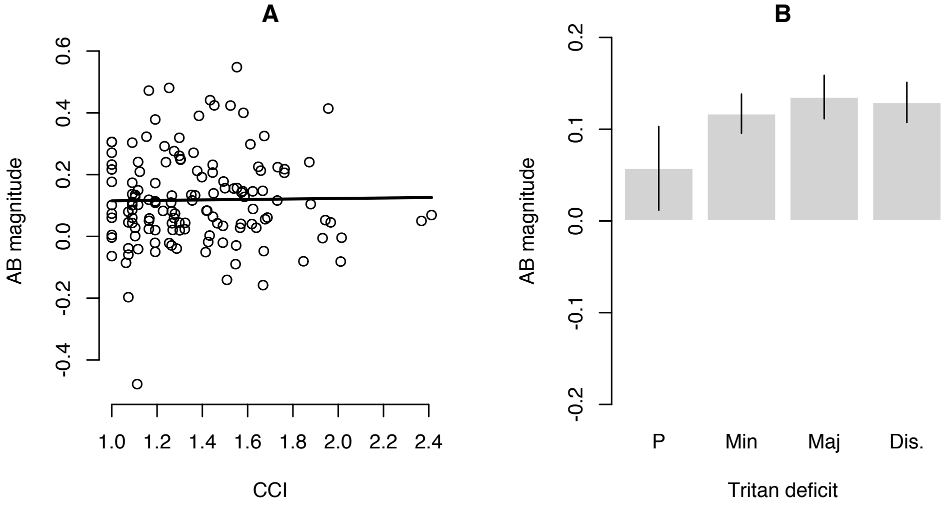

Figure S3. AB magnitude as a function of color discrimination performance for both data sets combined. A) Data does not indicate a relationship between the quantitative color discrimination variable (CCI) and AB size. B) Accuracy in color discrimination on the blue-yellow axis ('tritan deficit') does not explain AB magnitude differences Color discrimination on the blue-yellow axis was qualitatively assessed and participants were split up in groups accordingly; 'P' indicates perfect color discrimination; 'Min' and 'Maj' indicates minor and major errors in color discrimination respectively and 'Dis' reflects color discrimination on the blue-yellow axis that is classified as a disorder.

2.2.2.3. AB and mood

Polynomial regression analyses were carried out to test whether the AB magnitude is predicted by mood-related arousal and valence.

Analyses show that arousal before AB assessment (β = -.004, p=.622) does not predict the AB ($F(1,132)$= 0.24, p = .622, R^2 = -.005). Regression analysis for valence revealed that, in accordance

with arousal, the size of the AB is not significantly predicted by valence ($F(1,132)= 0.005, p=.940, R^2 = -.007$; β = -.001, $p=.940$).

2.2.3. Multiple regression model on DA markers

Multiple regression analysis was carried out to test whether the AB magnitude is predicted by all (continuous) explanatory variables tested, namely sEBR, color discrimination (CCI), and mood variables arousal and valence. Since, in the individual regression models did not reveal curvilinear relationships between the variables, the predictors are added as single, linear predictors.

Analyses show that the full model, including all continuous predictors, does not predict AB size ($F(15,118) =1.195, p= .284, R^2 = .02$). See table S1 for the beta estimates of the individual predictor terms.

Table S3. *Regression results using AB size as the criterion on pooled data.*

Predictor	ß	p-value
(Intercept)	-0.60	.869
sEBR	-0.16	.454
CCI	0.18	.947
arousal_ab	0.13	.837
valence_ab	0.21	.705
sEBR:CCI	0.14	.389
sEBR:arousal_ab	0.02	.547
CCI:arousal_ab	-0.03	.956
sEBR:valence_ab	0.02	.543
CCI:valence_ab	-0.11	.787
arousal_ab:valence_ab	-0.04	.661
sEBR:CCI:arousal_ab	-0.02	.456
sEBR:CCI:valence_ab	-0.02	.504
sEBR:arousal_ab:valence_ab	-0.00	.667
CCI:arousal_ab:valence_ab	0.02	.750
sEBR:CCI:arousal_ab:valence_ab	0.00	.610

3. AB – sEBR: experiment 2 – removal of influential observations

Experiment 2

As described in 3.2.2.1, polynomial regression in experiment 1 and 2 did not indicate any (linear or quadratic) relationship between AB and sEBR (Experiment 1: $F(1,69)=0.22, p=.641, R^2 =.003$; Experiment 2: $F(1,63)=0.00, p=.993, R^2 = -0.016$; figure 4). Hence, sEBR did not significantly predict the size of the AB (β = 0.000, $p=.993$). However, analyses of model fit of experiment 2 revealed that removal of influential observations in the regression model resulted in improvement of

the model. Although the model did not become significant and R^2 remained around zero, we here report the regression results of experiment 2 after removal of influential observations.

After removal of the most influential outlier, as suggested by the cook's distance (cook's distance = 0.45; 4 times bigger than the mean cook's distance), the relationship between AB and sEBR remained non-significant ($F(1,62)=0.00$, $p=.442$, $R^2 = -0.016$). Therefore, in our data sEBR did not significantly predict the size of the AB ($\beta = -\ 0.002$, $p=.442$).

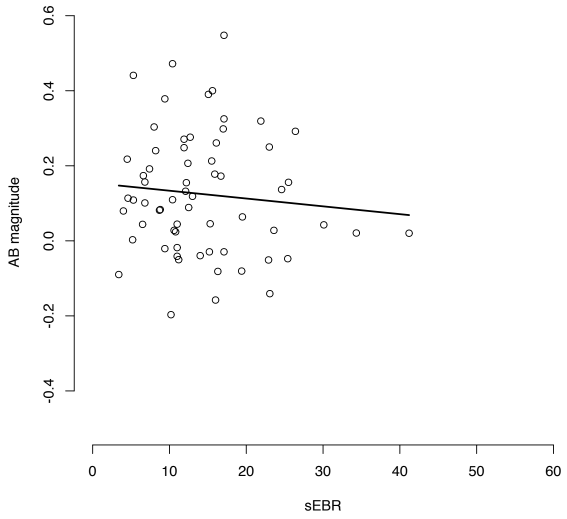

Figure S4. Experiment 2: sEBR did not significantly predict the size of the AB ($\beta = -.002$, $p=.442$) after removal of the most influential outlier (cook's distance = 0.45); $F(1,62)=0.00$, $p=.442$, $R^2 = -0.016$).

4. AB – sEBR: analysis with adjusted sEBR range

As our sEBR range (experiment 1: Range = [3.4 - 53.6]; experiment 2: Range = [1.8 - 63.4]) was a lot wider than the ranges reported in Colzato et al. (2008; Range = [2.4 – 31.8]) and Slagter & Georgopoulou (2013; Range = [5 – 35]), we wanted to make sure that our findings are not due to the extreme sEBR values. Accordingly, we re-analyzed the data of both experiments by cutting off those sEBR values that did not fall into the range of Colzato et al. (2008). We chose this range, as it is very similar to the sEBR range in Slagter & Georgopoulou (2013), and because Colzato and colleagues (2008) did find a significant correlation between the AB size and sEBR using this range. For experiment 1, also one AB outlier (AB magnitude < -0.4) was removed in order to minimize the

deviations in AB range to Colzato et al (2008). This resulted in a sample size of N=67 for experiment 1 and N=62 for experiment 2.

Experiment 1

Adjusting sEBR (and AB) range according to Colzato et al. (2008) did not reveal a relationship between AB and sEBR (F(1,61)= .002, p= .067, $R^2 < 0.001$). Data from experiment one does not indicate that sEBR significantly predicts the size of the AB ($\beta = 0.001$, p= .967).

Experiment 2

Adjusting sEBR (and AB) range according to Colzato et al. (2008) in experiment 2 did not show any relationship between AB and sEBR (F(1,60)= .095, p= .76, $R^2 = -0.015$). Therefore, data from experiment 2 also does not indicate that sEBR significantly predicts the size of the AB ($\beta = -0.001$, p= .76).

5. AB – sEBR: other AB measures (as in Willems et al., 2015)

As there are other ways to compute the size of the attentional blink besides the method used by Colzato et al. (2008) and Slagter & Georgopoulou (2013), we additionally tested the association between sEBR and AB size when AB magnitude is computed based on Willems et al. (2015). In this method, AB magnitude is calculated as a function of T1 accuracy. Please note that we had to adjust the formula from Willems et al. (2013) as we did not collect data at lag 2. Specifically, in our case, AB magnitude was computed for each individual by calculating the percentage decline in T2|T1 accuracy at lag 3 relative to T1 accuracy across lags.

As for the AB size presented earlier in the paper, computed based in Colzato et al. (2008), there was no correlation between AB size and sEBR in both experiments (Experiment 1: $r_s = .11$, p= .373; Experiment 2: $r_s = -.014$, p= .919).

6. AB: reversal ratio influences, an additional explorative analysis

Flexibility is associated with higher levels of striatal dopamine, which is according to the model described in the Introduction of the main manuscript believed to facilitate parallel processing by distributing attentional resources in such a way that it is allowing more information to be transferred into, and consolidated in, working memory. Yet, this distribution of attentional resources onto multiple items comes at a cost, as it enables more noise to enter working memory. One possible result of a more parallel processing style might be the loss of information on order of the presented

targets, by means of parallel processing to a degree that results information integration. As an 'integration' score, the ratio of reported targets in reversed order ('reversal ratio') was investigated. Accordingly, only trials were taken into account in which both T1 and T2|T1 were reported correctly.

First, we hypothesized that the reversal ratio is largest at lag 1. This would indicate that targets are processed parallelly when presented in short succession compared to when there is time to process each item independently. Secondly, we expected that individuals with a high reversal ratio would perform better at short lags compared to that individuals with a high reversal ratio, reflecting more successful integration of information (in this case targets). And finally, we hypothesized that a high reversal ratio is associated with smaller attentional blink, as it would suggest that the reversal ratio reflects the degree of parallel and flexible processing.

Experiment 1

In a first step, in order to investigate the influence of reversal ratio on the attentional blink, repeated-measures ANOVA (as in '2.4 Statistical analyses', paragraph 2) on accuracy were conducted with lag as within-subject factor with three levels (lag 1, lag 3, and lag 8). In a second step, the same analysis was repeated including a reversal ratio group factor based on a median split. In a third step, Spearman correlation tests were performed between reversal ration and sEBR, following our correlations to investigate replicability of the link between AB and sEBR, as also found in Colzato et al. (2008).

Analysis of the effect of lag on the reversal ratio revealed that there is a main effect of lag on the reversal ratio ($F(2, 140)= 306.91$, $p_{gg}<. 001$), which is largest at lag 1 (figure S5a). In order to test whether individuals with a high reversal ratio would perform better at short lags compared to that individuals with a high reversal ratio, we median split the data based on the mean reversal ratio. Indeed, there was a main effect of group ($F(2, 69)= 15.54$, $p <. 001$) and lag ($F(2, 138)= 41.98$, $p_{gg}<. 001$), but no interaction between reversal group and lag ($F(2, 138)= 0.37$, $p_{gg} = .658$). However, the direction of the group effect was opposite to our expectation (figure S5c), with high reversal ratios reflecting lower accuracy scores compared to low reversal scores. There was no significant correlation between AB size and reversal ratio ($r_s = -0.176$, $p= .143$).

Experiment 2

Data of experiment 2 revealed similar results as experiment 1. First, there is a main effect of lag on the reversal ratio ($F(2, 128)= 235.90$, $p_{gg}<.001$), with lag 1 significantly differing from lag 3 ($p< .001$) and lag 8 ($p< .001$; figure S5b). The reversal group analysis confirmed results from experiment 1: There was a main effect of reversal group ($F(2, 126)= 12.94$, $p=.006$; figure S5d) and lag ($F(2, 138)= 30.73$, $p_{gg}<.001$), but no interaction between reversal group and lag ($F(2, 126)= 1.07$, $p_{gg}=.348$). Again, there was no significant correlation between AB size and reversal ratio ($r_s = -0.019$, $p=.883$).

Taken findings of both experiments together, the reversal ratio does not seem to reflect an 'integration' score indicating a higher degree of parallel processing, as this would suggest smaller AB's and higher accuracy scores, especially on short lags, for 'high integraters'.

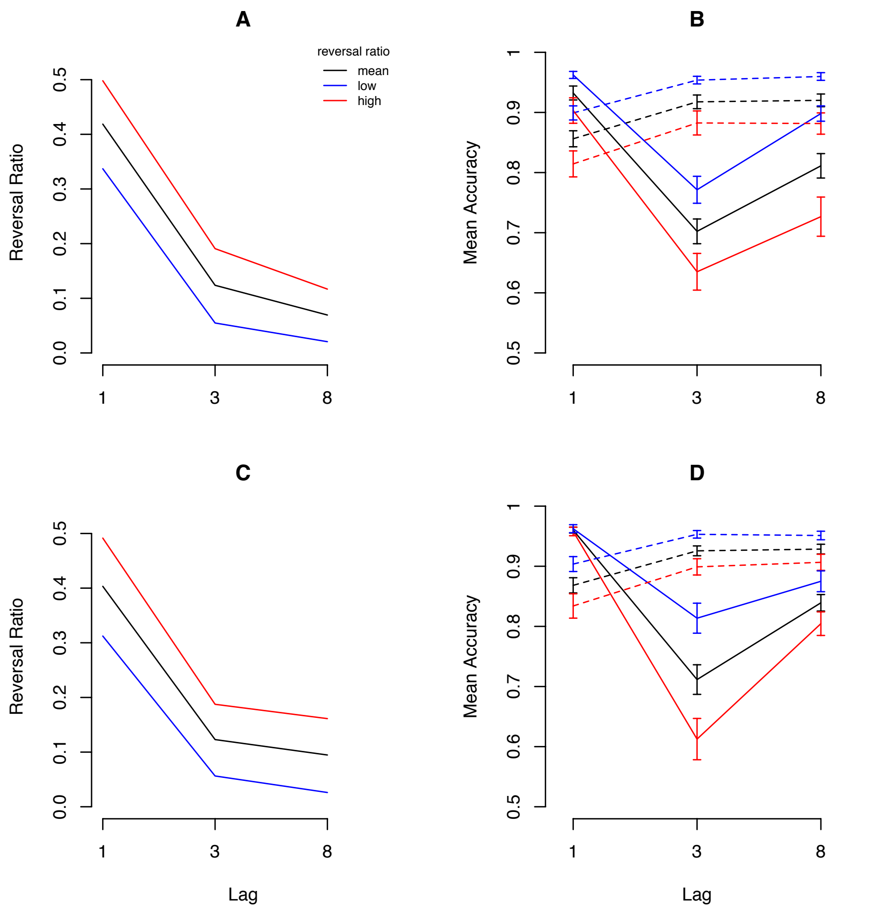

Figure S5. Reversal ratio effects on lag (A,C) and accuracy (B,D). Analysis of the effect of lag on the reversal ratio revealed that there is a main effect of lag on the reversal ratio with largest effects at lag 1 (Experiment 1: $F(2, 128)= 235.90$, $p_{gg}<.001$; Experiment 2: $F(2, 140)= 306.91$, $p_{gg}<.001$). Analysis of reversal groups (based on median split mean reversal ratio) showed a direction of the group effect that was opposite to our expectation (Experiment 1: $F(2, 69)= 15.54$, $p<.001$; Experiment 2: $F(2, 126)= 12.94$, $p= .006$).

7. sEBR and mood

Given findings that mood and sEBR are correlated, suggesting an association between mood and this non-invasive proxy of striatal dopamine, we additionally tested whether we can replicate this finding shown in Akbari Chermahini & Hommel (2012).

When combining both data sets, analysis reveal a negative correlation between sEBR and arousal (r_s = -.18, *p*= .037), but not between sEBR and valence (r_s = -.11, *p*= .213). However, with very low effect sizes.

In experiment 1, arousal negatively correlates with sEBR (r_s = -.293, *p*= .015) while valence is also negatively, but not significantly, associated with sEBR (r_s = -.220, *p*= .069). Data from experiment 2 however cannot confirm the effects seen in experiment 1, as the correlation was not significant for both, arousal (r_s = -.042, *p*= .739) and valence (r_s = .013, *p*= .913).

Taken together, our findings show a small relationship between arousal and sEBR but failed to show a relationship between valence and sEBR. The results suggest that, in contrast to Akbari Chermahini & Hommel (2012), only the arousal level, but not the 'direction' of mood (positive versus negative) seems to relate to the sEBR. One reason for the failure to fully replicate the finding from Akbari Chermahini & Hommel (2012) could be the different instruments used to assess mood (PANAS in Akbari Chermahini & Hommel, 2012, and the affect grid in our study). Second, while Akbari Chermahini & Hommel (2012) focused on changes in sEBR due to mood induction, we only correlated mood scores with sEBR at the time of mood measurement and therefore did not monitor changes in either of the scales.
